# Functional Recovery of AQP2 Recessive Mutations Through Hetero-Oligomerization with Wild-Type Counterpart

**DOI:** 10.1038/srep33298

**Published:** 2016-09-19

**Authors:** Abdulah El Tarazi, Yoann Lussier, Sandra Da Cal, Pierre Bissonnette, Daniel G. Bichet

**Affiliations:** 1 Groupe d’Étude des Protéines Membranaires (GÉPROM), département de Physiologie moléculaire et intégrative, Université de Montréal, C.P. 6128, Succ. Centre-Ville, Montréal, Québec, H3C 3J7, Canada; 2 Centre de Recherche, Hôpital du Sacré-Cœur de Montréal, Montréal, Québec, H4J 1C5, Canada

## Abstract

Aquaporin-2 (AQP2) is a homotetrameric water channel responsible for the final water reuptake in the kidney. Mutations in the protein induce nephrogenic diabetes insipidus (NDI), which challenges the water balance by producing large urinary volumes. Although recessive AQP2 mutations are believed to generate non-functional and monomeric proteins, the literature identifies several mild mutations which suggest the existence of mixed wt/mut tetramers likely to carry function in heterozygotes. Using *Xenopus* oocytes, we tested this hypothesis and found that mild mutants (V24A, D150E) can associate with *wt*-AQP2 in mixed heteromers, providing clear functional gain in the process (62 ± 17% and 63 ± 17% increases, respectively), conversely to the strong monomeric R187C mutant which fails to associate with *wt*-AQP2. In kidney cells, both V24A and D150E display restored targeting while R187C remains in intracellular stores. Using a collection of mutations to expand recovery analyses, we demonstrate that inter-unit contacts are central to this recovery process. These results not only present the ground data for the functional recovery of recessive AQP2 mutants through heteromerization, which prompt to revisit the accepted NDI model, but more importantly describe a general recovery process that could impact on all multimeric systems where recessive mutations are found.

The fine-tuning of body water homeostasis is performed by controlling Aquaporin-2 (AQP2) activity in principal cells of the collecting tubule, a task under the control of the antidiuretic hormone vasopressin^1^,^2^,^3^,^4^. Mutations in this water channel result in nephrogenic diabetes insipidus (NDI), a condition where episodes of polyuria uncompensated by polydipsia may lead to dehydration with corollary health problems and possibly death^5^,^6^. Abundant data describing the key features of mutated AQP2 forms have led to a general model for AQP2-dependent NDI which centers on subunit interactions within the homotetrameric structure of the channel. According to this scheme, recessive (*rec*) mutations are believed to produce ill-structured proteins unable to oligomerize, hence inducing NDI phenotypes in *rec/rec* homozygotes where only non-functional monomeric AQP2 particles are expressed^7^. Conversely, dominant (*dom*) AQP2 mutations retain their ability to associate with *wt*-AQP2 and thus induce NDI phenotypes even in *wt/dom* heterozygotes through sequestration of heteromeric complexes (*dominant negative effect*)^1^,^8^.

Yet, several *rec*–AQP2 mutants of mild phenotypes displaying partial plasma membrane targeting and functionality have been reported^7^,^9^,^10^,^11^,^12^,^13^, which clearly revoke the notion that all are ill-structured, and consequently challenge the proposed model for NDI^14^. More importantly, if *rec*-AQP2 mutations do functionally multimerize, the possibility for mixed *wt/rec* heteromers becomes even more probable with *wt*-AQP2 acting as support or molecular rectifier to recuperate *rec*-AQP2 forms. Consequently, unsuspected yet functional *wt*/*rec* heteromers may well be of regular occurrence and not an exception, which would reduce the deleterious impact of the mutation through overall increased functionality in heterozygous individuals.

With this premise, we have investigated the protein-protein relations within *wt*- and *rec*-AQP2 from a series of reported clinical cases, looking at both molecular (oligomerization state) and functional (water permeability) features to evaluate the possible recovery of such *rec-*AQP2 mutants. Our results not only show that *wt/rec* heteromers do exist, but we also confirm that this condition confers a clear increase in functionality through both stabilization, and increased plasma membrane targeting of the *rec*-AQP2 forms.

## Results

### Mild mutations can oligomerize

**Figure 1:**
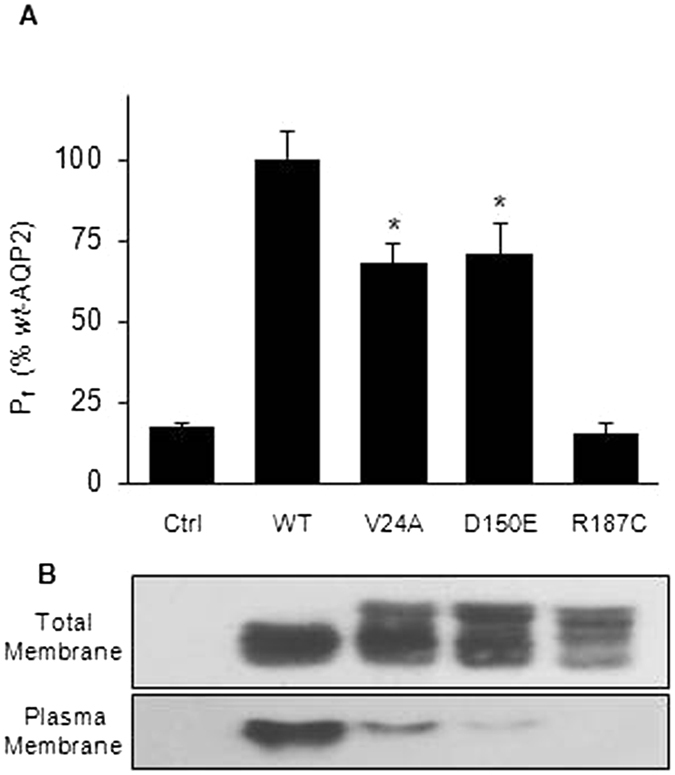
Comparative expression of AQP2 variants in oocytes. Oocytes were injected with mRNAs so to generate equivalent protein loads for each AQP2 variants (0.5 ng wt-AQP2, R187C, 1.5 ng V24A and 2.5 ng D150E) and incubated for 24 hours prior testing for water permeability (**A**) and Western blot (**B**) on both total and plasma membranes. Water permeability values (P_f_) are in % ± SD of *wt*-AQP2 with n = 8 per condition, and is typical of 5 assays. Asterisks indicate statistical significance in comparison to both water-injected (p < 0.001) and *wt*-AQP2 (p = 0.05) oocytes.

When evaluating *rec*-AQP2 in oocytes, expression levels of the protein and plasma membrane targeting are the key factors governing the overall functional outcome. This is exemplified in Fig. 1 where three mutants are compared to *wt*-AQP2. Here, RNA loads of 0.5 ng (*wt*-AQP2, R187C), 1.5 ng (V24A) and 2.5 ng (D150E) were required to generate equivalent amounts of AQP2 proteins, as typically performed for such assays^12^,^13^, stressing variations in protein stability (synthesis/degradation) amongst AQP2 mutations (Fig. 1B, total membranes). Plasma membrane targeting also varies, spanning from maximal efficiency (*wt*-AQP2) to complete lack of targeting (R187C), along partial capacities for both V24A and D150E (Fig. 1B, plasma membrane), which adequately correlate to respective functionality determined by water permeability evaluations (Fig. 1A; *wt*-AQP2 = 100%, V24A = 68 ± 6% and D150E = 71 ± 10%, n = 5 assays). As expected, R187C is non-functional, similar to water-injected oocytes (16 ± 3% and 18 ± 1% respectively). Consequently, V24A and D150E are considered to be mild mutations while R187C represent a severe NDI-causing mutation.

**Figure 2:**
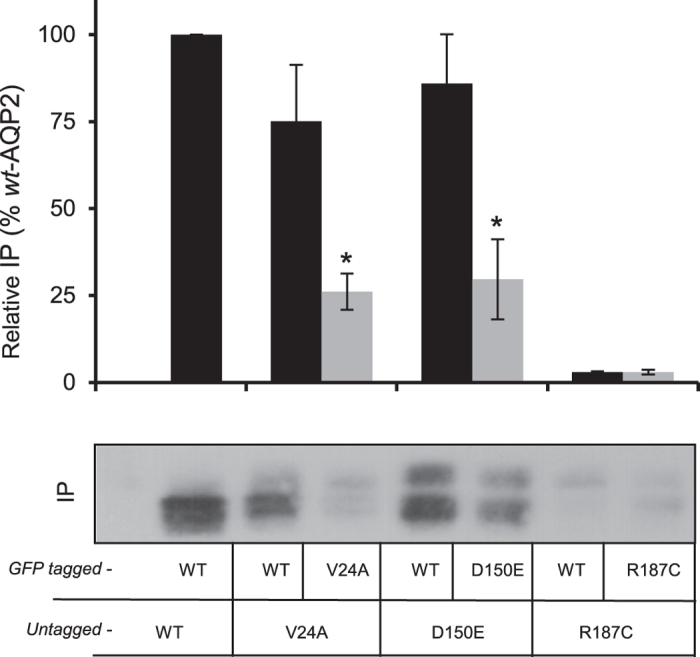
Immunoprecipitation of AQP2 in homozygous and heterozygous condition. Oocytes were injected with 0.5 ng mRNAs for each untagged AQP2 variants (*untagged*-WT, V24A, D150E and R187C) along equimolar amounts (1 ng) of corresponding GFP-tagged forms (*GFP-tagged*-WT, V24A, D150E and R187C) and incubated for 24 hours prior immunoprecipitation (IP) using anti-GFP (see methods). Blots were probed using anti-AQP2 and figure shows the pulled-down untagged variants (29 kDa) for every IP condition. Results are typical of 4 assays from which individual densitometry were performed and transposed in bar graph as % of *wt*-AQP2. Asterisks indicate statistical significance (p = 0.05) in comparison to corresponding + WT conditions.

The functionality of both V24A and D150E is indicative of their adequate molecular structures (tetramers), and further suggests the ability for such mild mutations to produce homomeric (*rec/rec*), and possibly heteromeric (*wt/rec*) tetramers, as is found with dominant mutations^15^,^16^. Consequently, we evaluated the capacity for each *rec*-AQP2 to multimerize either with self (*rec*/*rec*) or with *wt*-AQP2 (*wt/rec*), using a co-IP strategy where GFP-tagged AQP2 variants are used to pull-down (IP) untagged variants (see methods and^17^). In this assay, oocytes were injected with equimolar RNA loads, mimicking the diploid (homo/heterozygous) condition. The Western blot in Fig. 2 shows IP bands of the untagged AQP2 variants for each condition, along densitometry analysis to evaluate their propensity to multimerize. As shown in bar graph, the capacity for both V24A and D150E to associate as *rec*/*rec* homomers (grey bars: 26 ± 5% and 30 ± 11% of *wt*-AQP2, respectively) is increased 2.9 fold when paired with *wt*-AQP2 within *wt/rec* heteromers (black bars: 75 ± 16% and 86 ± 14% of *wt*-AQP2, respectively). Conversely, R187C fails to multimerize with self, as with *wt*-AQP2 (3 ± 0.9% and 2 ± 0.3% of *wt*-AQP2, respectively), as expected from a monomeric mutant.

### Mild mutations can be functionally recovered

**Figure 3:**
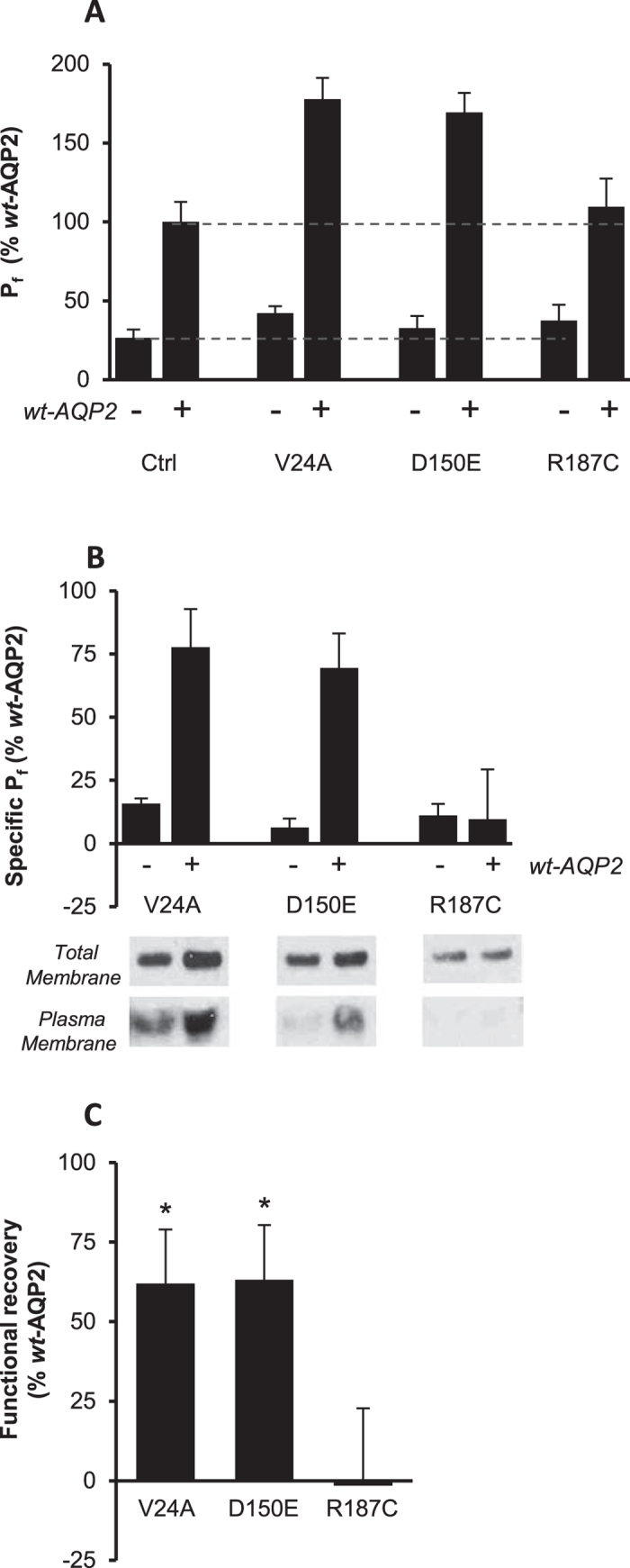
Functional recovery analysis for *rec*-AQP2 forms in oocytes. Oocytes were injected with 0.5 ng mRNA coding for *wt*, V24A, D150E or R187C either in absence (−) or presence (+) of same amount of *wt*-AQP2 and incubated for 24 hours prior testing for water permeability (P_f_). Activities are presented in % ± SD of *wt*-AQP2 with n = 8 per condition, and is typical of 6 assays. (**A**) Total activity. (**B**) Specific activity for single (control value subtracted from all (−) expressions), and double (*wt*-AQP2 value subtracted from all (+) expressions) expressing conditions. (**C**) Gain of function for each AQP2 variant calculated by subtracting specific P_f_ values determined in absence (−) to those determined in presence (+) of *wt*-AQP2. Western blots in panel B represent specific labeling (29 kDa) for each mutant either in absence (−) or presence (+) of GFP-*wt*-AQP2 (58 kDa, not seen in blot), for both total and plasma membranes. Asterisks indicate statistical significance from zero (p = 0.001).

Using biochemical and functional approaches, we then set to determine if *rec*-AQP2 forms could actually be functionally recovered through coexpression along *wt*-AQP2. To specifically evaluate the impact of *wt*-AQP2 on *rec*-AQP2 activity, oocytes were injected with RNA coding for each AQP2 variant (*wt*, V24A, D150E and R187C), either in presence (+) or absence (−) of *wt*-AQP2, and tested for water permeability, as shown in Fig. 3A. Specific activities for each variant were then determined by subtracting non-specific values (dashed lines); 1- control value (Ctrl −) from single (−) expressing conditions, and 2- *wt*-AQP2 value (Ctrl+) from dual (+) expression conditions, thus isolating activities for each mutant in both conditions (absence (−) and presence (+) of *wt*-AQP2: panel B). Here, a positive discrepancy between both expressing conditions, subtracting single (−) to double (+) expression values in panel B, is indicative of an effective gain in function induced by *wt*-AQP2 (panel C). The Fig. 3 presents a typical analysis showing total (panel A) and specific (panel B) activities to which effective gains of function were extracted (panel C: activity increases of 63.9 ± 23.2% and 65.2 ± 25.3% for V24A and D150E respectively, in % *wt*-AQP2 activity, n = 6 to 8 assays). In comparison, the monomeric R187C exhibits no specific activity in both single and coexpression conditions (panel B) and thus not functionally recovered (panel C; 0.4 ± 32.9%). Western blots in same expression conditions were found to adequately correlate with increased protein contents (total membranes) and/or plasma membrane targeting (plasma membranes). Again, GFP-*wt*-AQP2 (58 kDa) was used in coinjection (+) conditions so to isolate *rec*-AQP2 signals (29 kDa bands, shown in blots). As presented in Fig. 3B, protein abundances increased for both V24A (2.8 ± 0.5 fold) and D150E (2.2 ± 0.2fold) when coexpressed along *wt*-AQP2 (total membrane fractions; average densitometry for 3 assays). Conversely, R187C (1.6 ± 0.5fold) is not affected by the presence of GFP-*wt*-AQP2. Similar analyses performed on purified plasma membranes also showed increased bands for both V24A and D150E when in presence of GFP-*wt*-AQP2 (+) while *wt*-AQP2 is unchanged. As expected, R187C remained absent from plasma membrane (as in Fig. 1), even in the presence of GFP-*wt*-AQP2.

**Figure 4:**
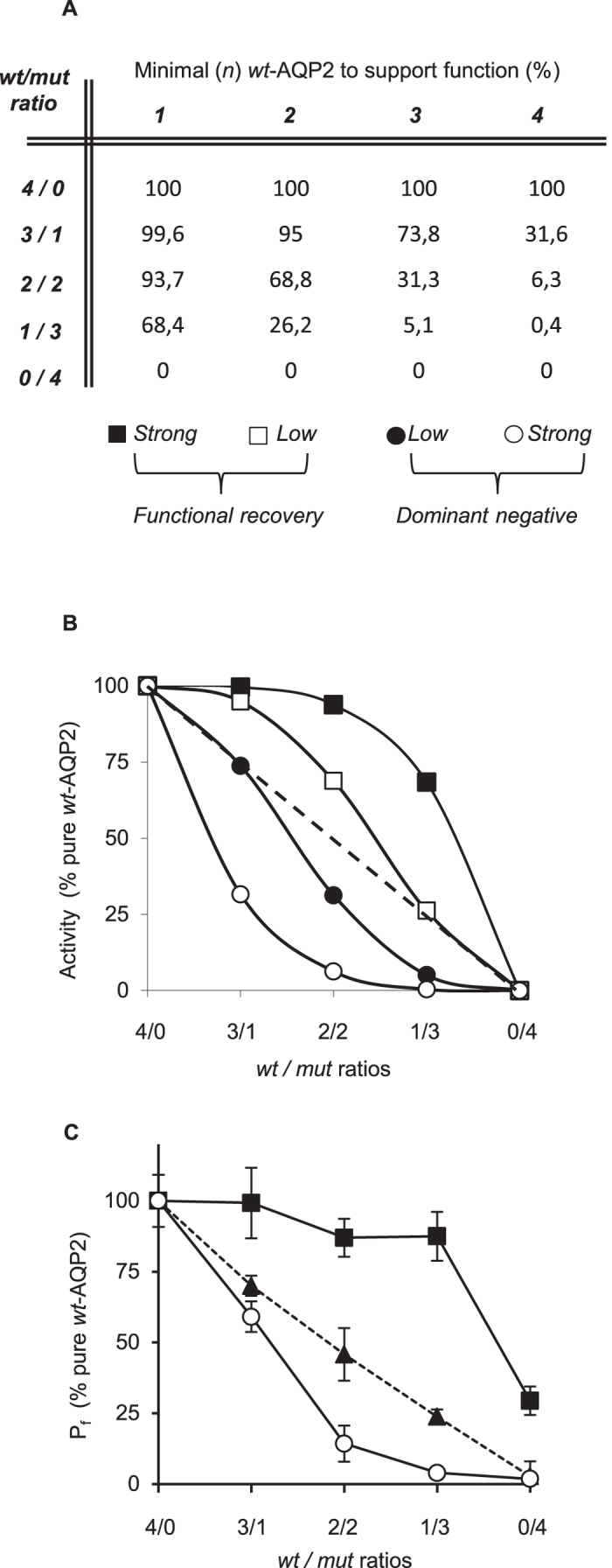
Functional recovery analysis in function of varying *wt/mut* ratios. (**A**) Theoretical model describing activity projections (% pure *wt*-AQP2) for varying *wt/mut* ratios (4/0 to 0/4) when assuming a minimal number of functional units within the tetramer to allow activity (*n wt*-AQP2 = 1 to 4). (**B**) Transposition of theoretical data, plotting activity levels against *wt/mut* ratios for each minimal requirement (*n* = 1 (■), 2 (□), 3 (●) or 4 (○)). Dash line represent non-interacting condition. C) Oocytes were injected with 1 ng mRNA mixtures of varying *wt/mut* ratios (4/0 to 0/4) combining *wt*–AQP2 to R254Q, D150E or R187C and incubated for 24 hours prior testing for water permeability. Activities (P_f_) are presented in % ± SD of pure *wt*-AQP2 with n = 8 per condition and is typical of 3 assays. As shown, R254Q display typical dominant negative effect while D150E is compatible with strong functional recovery. As expected, R187C does not affect *wt*-AQP2 function, in accordance with its non-interfering (monomeric) nature.

The key distinction between *functional recovery* and *dominant negative effect* resides in the fate of the mixed heteromers, either producing an effective recuperation (*wt/rec*) or intracellular retention (*wt/dom*). In that regard, a statistic model can be developed describing theoretical activity profiles for both phenomena. As shown in Fig. 4A, a classical binomial distribution of *wt/mut* combinations was used to generate theoretical activity projections as a function of the minimal *wt/mut* structure required to support channel activity (*n*/4 *wt*-AQP2 moieties). Here, the model developed used 4:0 to 0:4 ratios (*wt-* to *mut-*) so to conveniently parallel the tetrameric structure of AQP2. Figure 4B recapitulates the data and describes the propensity for a tetramer to be inhibited (dominant mutations; lower curves (○,●)) or not (recessive mutations; higher curves, (□,■)) by its mutated constituents. Accordingly, coexpression with a non-interacting mutation will only generate a linear decrease in function (dashed line), reflecting the fractional expression of the *wt*-AQP2 constituent.

Using key mutations attributed to dominant negative (R254Q^18^), non-interacting (R187C) and functional recovery (D150E) behaviors, we have challenged these projections using the same *wt*- to *mut*- ratios (4:0 to 0:4 ratios) in total RNA loads of 1 ng/oocytes, so to prevent any competition in protein synthesis^15^. The Fig. 4C confirms the projections showing not only the dominant negative action of R254Q (compatible with strong dominant negative profile, ○) but more importantly the high recuperation of recessive V24A (compatible with strong recovery profile, ■). As expected, coexpression along R187C produces activity levels reflecting that of *wt*-AQP2. Of high interest is the 2/2 ratio representing the heterozygous condition (2/2 = 1:1 ratio = *wt/mut* heterozygote) which is found to be the most discriminatory with theoretical activity spanning from 6% to 94% (from strong inhibition to strong recovery, respectively, panel B) and incidentally found herein for both mutant tested (panel C; 14 ± 6% to 87 ± 7% for R254Q and V24A respectively).

### Testing recovery with other *rec* mutants

**Figure 5:**
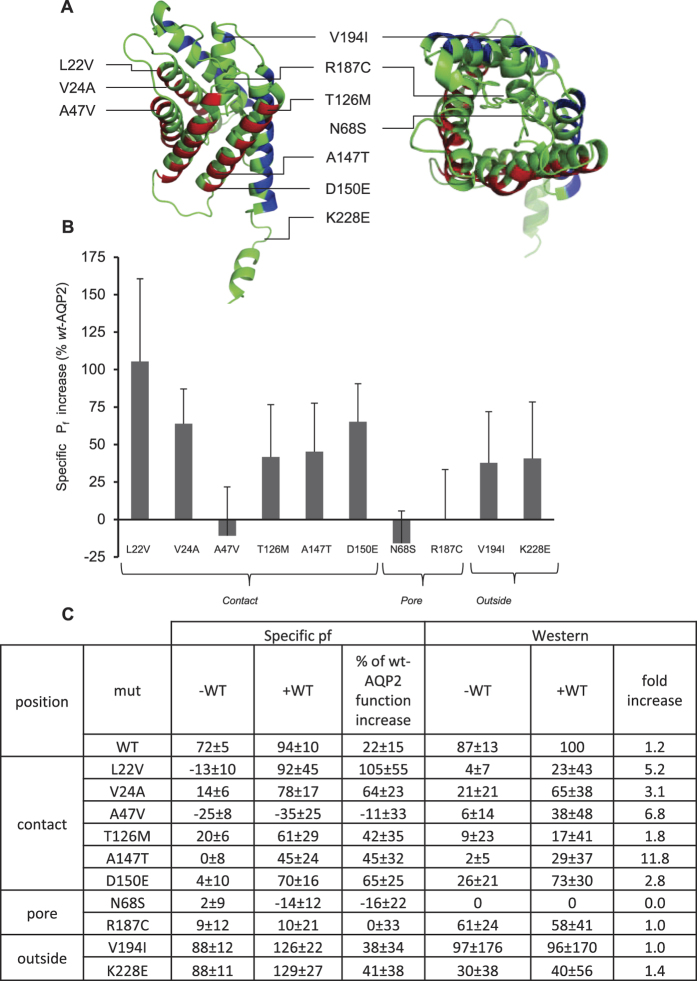
Functional recovery of known *rec*-AQP2 located in distinct areas of the protein. Oocytes were injected with 0.5 ng mRNA coding for *wt*, L22V, V24A, A47V, T126M, A147T, D150E, N68S, R187C, V194I or K228E either in absence (−) or presence (+) of same amount of *wt*-AQP2 and incubated for 24 hours prior testing for water permeability (see Fig. 3). Activities (P_f_) are presented in % ± SD of *wt*-AQP2 with n = 3 to 8 per condition, combining 6 assays. (**A**) Location of the mutations within the reported structure of AQP2 (33) (red = interunit faces, blue = exofacial faces). (**B**) Histogram presenting gain of function ± SD for each AQP2 variant, as in Fig. 3C. (**C**) Table presenting specific activities (P_f_) along Western bands densitometry (Western) for each mutant in both absence (−WT) and presence (+WT) of *wt-*AQP2.

We expanded this study using previously reported mutations chosen according to their positions within the AQP2 structure (interunit contact, pore, and external locations; see Fig. 5A). Results show that mutations providing inter-unit contact (L22V, V24A, T126M, A147T and D150E) show significant gain of function when expressed along *wt*-AQP2 (net increases of 106 ± 56%, 64 ± 23%, 42 ± 35%, 45 ± 32% and 65 ± 25% of the *wt*-AQP2 activity, respectively; Fig. 5B,C), with noticeable exception of A47V (−11 ± 33%), while mutations associated with the pore structure (N68S and R187C) show no significant gain of function in same condition (−16 ± 22% and 0 ± 33%, respectively). Finally, mutations located at the periphery of the AQP2 tetrameric structure (V194I and K228E), which are somewhat active in oocytes even in absence of *wt*-AQP2 (88 ± 12% and 88 ± 11% of *wt*-AQP2 activity), display no specific gain of function when expressed with wild type counterpart (38 ± 34% and 41 ± 38% respectively). These results are mirrored by density analysis in Western blots which show specific band increases when in presence of *wt*-AQP2 only for contact mutations (2.8 to 11.8 fold), in comparison to pore and peripheral mutants (0 to 1.4 fold).

### Functional recovery in cell lines

Finally, we tested the recovery of plasma membrane targeting for *rec*-AQP2 using the mpkCCD_c14_ cell model^19^,^20^. While varying mRNA loads is a typical approach to compare AQP2 mutants (Fig. 1), evaluating interactions within *wt*- and *mut*-AQP2 forms so to mimic true heterozygous or homozygous conditions commands the use of equivalent mRNA loads. Although readily achievable in oocytes (mRNA injections), the same cannot be said with dual transfections using cell line models. For that reason, we have opted for a bidirectional expression system which provides with strict equivalent mRNA expressions for both inserts within each transfected cells (1:1 mRNA ratios; see methods).

**Figure 6:**
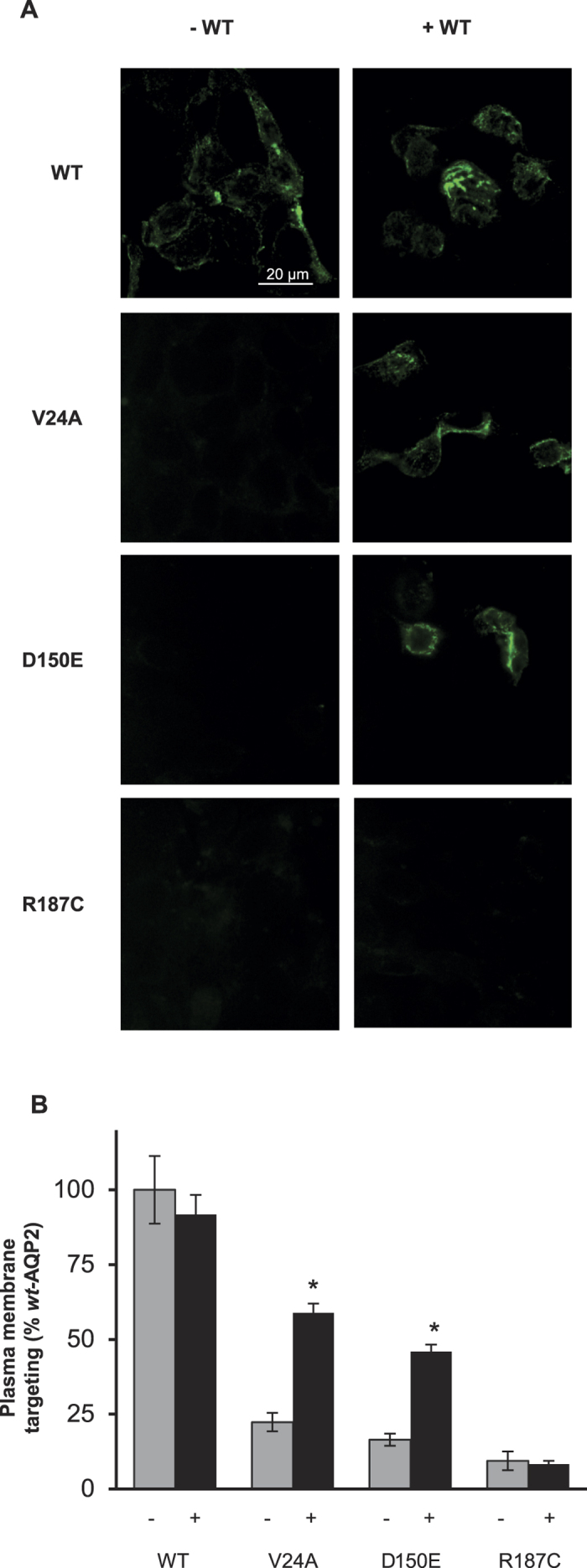
Functional recovery of *rec*-AQP2 forms in mpkCCD_c14_ cells. Cells grown on semipermeable filters were transfected with pBi vectors expressing FLAG-tagged AQP2 variants (V24A, D150E and R187C) as unique transcript (−) or along untagged *wt*-AQP2 (+) as second transcript (see methods). Immunofluorescence using anti-FLAG allowed visualization of tagged variants to evaluate effective apical membrane targeting. (**A**) Induction of plasma membrane targeting for V24A and D150E, but not R187C, by coexpression with *wt*-AQP2. (**B**) Histogram of positive apical membrane targeting for each condition, presenting mean ± SD of cell count in a 60x field + 8 adjacent fields, for 3 assays. Asterisks indicate statistical significance from -WT condition. (p < 0.001).

Cells were thus transfected using bidirectional vectors encoding each AQP2 variants (exofacial FLAG-tagged), either alone (single insert) or along untagged *wt*-AQP2 (double inserts), and tested the next day to evaluate the apical targeting of the FLAG-tagged variants through positive immunofluorescence labeling (cell counts of 9 adjacent fields, values in % of *wt*-AQP2, see methods). Transfections using single insert vectors (Fig. 6A) essentially displayed little to no plasma membrane targeting for all mutant forms (22 ± 5%, 16 ± 4% and 9 ± 2% for V24A, D150E and R187C, respectively). In presence of *wt*-AQP2 (double inserts vectors), positive labeling were enhanced almost threefold for both V24A (58 ± 5%) and D150E (46 ± 3%), consistent with targeting recovery found in oocytes (Fig. 3B). Again, R187C remained within intracellular stores (8 ± 5%). The histogram presented in Fig. 6B represents the overall statistic sampled within 3 experiments.

## Discussion

Most NDI-causing AQP2 mutations identified so far were classified as recessive in accordance to genotypes/phenotypes analyses. Structurally, these are distributed throughout the AQP2 structure with exception of the intracellular C-terminus, which encompass all dominant mutations. Although presented as monomeric, intracellularly sequestered, ill-formed and non-functional proteins^21^,^22^,^23^,^24^, *rec*-AQP2 mutants nonetheless often display partial functionality with single channel permeability values similar if not identical to *wt*-AQP2^10^,^13^,^23^,^25^,^26^. Such activities, indicative of competent tetrameric molecular structures, clearly contradict the basic model for recessive NDI and opens to the possibility of naturally existing *wt/rec* heteromers. This study aimed at validating this concept, questioning its means and functional impact for NDI.

Suggesting functional recovery of *rec*-AQP2 mutations through *wt*-AQP2 oligomerization is unconventional and requires solid substantiation. Currently, it is believed that only dominant AQP2 mutations can achieve heteromeric (*wt/mut*) associations^14^,^27^. But this concept could be misleading as parallel studies performed on *rec*-AQP2 have essentially relied on R187C, a null mutant of known monomeric structure^8^,^14^, clearly not representative of the diversity found within mild *rec*-AQP2 mutants.

Consequently, we initiated this study using *rec*-AQP2 candidates presenting with partial functionalities susceptible to be improved, selecting the previously reported V24A and D150E variants^10^,^11^. Such mild phenotypes are here again confirmed in Fig. 1 (reduced expression, targeting and functionality), thus validating both mutations for recovery assays. In comparison, the lack of targeting and functionality of R187C corroborate its choice as negative control.

The co-IP data presented in Fig. 2 show that both V24A and D150E can multimerize to form homotetramers (densitometry; 26 ± 5% and 30 ± 11% of *wt*-AQP2, respectively) which, to our knowledge, has never been reported before. More important though is the fact that this capacity is enhanced threefold when paired to *wt*-AQP2 within heterotetramers (Fig. 2; 75 ± 16% and 86 ± 14% of *wt*-AQP2 for V24A and D150E, respectively). As this behaviour is also found in activity assays (P_f_, Fig. 3), we suspect the functional recovery process of *rec*-AQP2 to result from hetero-oligomerization to *wt*-AQP2 moieties, inducing stabilization and targeting of the mutant subunit(s) within *wt/rec* complexes, and providing protection of the recovered *rec*-AQP2 moieties from premature degradation. Taken together, these results suggest an effective rectifier role for *wt*-AQP2 over (at least some) mild recessive mutations. Conversely, the severe R187C mutation fails to display any sign of recovery when in presence of *wt*-AQP2 (Figs 2 and 3: lack of increased functionality, protein stabilization or effective plasma membrane targeting), again in line with previous data and consistent with its monomeric structure^8^,^14^,^15^.

To further this investigation, we extended our analysis using several known recessive mutations and found that those participating in interunit contacts are more prone to functional recovery, globally correlating protein stabilisation to increased activity features (L22V, V24A, A47V, T126M and D150E, with notable exception of A47V; see Fig. 5). These data further support the notion that the recovery process of *rec*-AQP2 results from an improved or restored interunit linkage within *wt/rec*-AQP2 heteromers, protecting the mutant moieties from premature degradation. On the other hand, mutations located in the pore are either not expressed (N68S) or non-functional (R187C), and fail to be recuperated when coexpressed along *wt*-AQP2. Finally, mutations located on the outskirts of the protein (V194I and K228E) already display robust functionality in oocytes and thus not influenced by *wt-*AQP2 coexpression.

Although *Xenopus* oocytes generate key data describing AQP2 mutation properties, studies using relevant models such as cell lines remain essential to validate the NDI phenotype, especially when targeting features are questioned^28^. This is exemplified with several *rec*-AQP2 forms which display activity in oocytes (K228E, V24A, and P262L) but fail to adequately traffic in mammalian cells (IMCD)^10^,^25^,^29^. The recovery of targeting properties for both V24A and D150E in a kidney cell model (mpkCCD_c14_), as shown in Fig. 5, was thus a critical step to ultimately credit this behaviour with physiological relevance.

The 1:1 *wt/mut* ratio was shown herein to be the most discriminant condition to characterize mutant forms with activity values spanning from 6% to 94% (strong inhibition to strong recovery, respectively, Fig. 4B), a feature which clearly emphasizes the duality and discrepancy opposing dominant negative effect to functional recovery behavior. This is exemplified in Fig. 4C in which V24A displays a strong recovery capacity (87 ± 7% of *wt/wt*) suggesting that a single *wt*-AQP2 moiety is sufficient to recover adequate function of the tetramer (*n* = 1 model), while the strong inhibition induced by R254Q (14 ± 6% of *wt/wt*) rather suggests that the presence of a single mutant subunit within the tetramer is sufficient to inhibit function (*n* = 4 model).

Most important is the knowledge that recessive mutations are not by definition all ill-formed, making the contribution of *wt/rec* tetramers to be considered in heterozygotes. In fact, we expect the recovery of *rec*-AQP2 forms demonstrated herein *in vitro* to also take place in heterozygotes, improving overall AQP2 function in NDI bearers through improved activity and targeting, which could be clinically relevant when challenging conditions prevail, such as water deprivation environments. The functional recovery principle, demonstrated herein in the context of AQP2 and NDI aetiology, describes a new class of recessive mutations recoverable through wild-type heteromerization. It is a new paradigm which mirrors the dominant negative effect, and consequently one to be considered with all multimeric systems where recessive mutations are found.

## Material and Methods

### Vectors and cRNA

D150E, V24A and R187C mutations were generated using site-directed mutagenesis on the pT7Ts vector, as previously described^10^,^11^. To generate pT7Ts-GFP-AQP2, the human AQP2 sequence was first subcloned form pT7Ts-AQP2 into the pAc-GFP vector (Invitrogen), producing the pAc-GFP-AQP2 vector. BclI and SpeI restriction sites were then added at flanking 5′ and 3′ ends of GFP-AQP2 coding sequence then subcloned back into pT7Ts vector to produce pT7Ts-GFP-AQP2. Vectors were linearized with SalI and cRNAs synthesized using the mMessage mMachine T7 kit (Ambion, Austin, TX). For expression in mpkCCD_c14_ cells, all constructs were performed using the double cistronic pBi-CMV1 vectors (Clontech) so to impose a tight synchronization for both transcripts expression within each transfected cell. First, FLAG-tagged AQP2 (wild-type and mutants) were subcloned into the MCS1 of pBI-CMV1 vector using NheI and SalI restriction sites. Then, when required (double expressions), untagged *wt*-AQP2 was subcloned from pDNR-1-*wt*-AQP2 (clontech) into the MCS2 of pBI-CMV1 vector using EcoRI and XbaI restriction sites.

### Oocyte preparation, injection and maintenance

Detailed preparation of oocytes was described previously^10^. Briefly, mature oocytes from gravid *Xenopus laevis* frogs were dissected by hand and treated with collagenase (17.5 mg/ml, Type 1A, Sigma-Aldrich) in a Ca^++^ free Barth’s solution (in mM: 90 NaCl, 3 KCl, 0.82 MgSO_4_, and 5 HEPES pH 7.6) to remove follicles. When required, euthanasia of animals was performed by prolonged anaesthesia. The oocytes were thereafter kept at 18 °C in normal Barth’s solution (same as above with 0.4 mM CaCl_2_ and 0.33 Ca(NO_3_)_2_) supplemented with horse serum (5%), sodium pyruvate (2.5 mM) and antibiotics (100 U/ml penicillin, 0.1 mg/ml streptomycin and 0.1 mg/ml kanamycin). The oocytes were then injected using a microinjection apparatus (Drummond Scientific, Broomall, PA) with 46 μl of water (controls) or AQP2 cRNA solutions to meet the required amount (see figure legends) and further incubated for 18–24 hours before experimentation.

### Cell culture maintenance and Transfection

mpkCCD_c14_ cells were routinely cultured in DMEM-F12 media supplemented with 10% FBS and antibiotics (100 U/ml penicillin and 100 μg/ml streptomycin) and maintained in a 95% air 5% CO_2_ atmosphere. For immunocytochemistry assays, cells were seeded on permeable filters in the same culture media and transfected the next day (60–90% confluency) using Lipofectamine 2000 (Invitrogen) according to the manufacturer, and further incubated for 24 hours before experimentation.

### Preparation of total and plasma membrane fractions of oocytes

For descriptive methodology, see^30^. Briefly, total membranes were prepared by homogenizing 15 oocytes in 1 ml PBS then centrifuged at low (250 × g for 5 min.) and high (20,000 × g for 20 min.) speed. Final pellets were resuspended in 30 μl PBS (2 μl solution/oocyte) and processed immediately for Western blots or immunoprecipitation. For purification of plasma membranes, 40 oocytes were treated for 10 minutes at room temperature with 0.005% subtilisin A (Sigma-Aldrich, #P5380) diluted in MBSS (80 mM NaCl, 20 mM MES pH 6.0) followed by two 1 hour polymerizing steps consisting first in 1% ludox (Sigma-Aldrich, # 420883), then with 0.1% polyacrylic acid; (Sigma-Aldrich, MW 30,000 # 41.604-5) again diluted in MBSS and performed on ice. The oocytes were then homogenised in 1 ml cold HbA using a P200 pipettor then subjected to successive low speed centrifugations (16, 16, 25 and 35 × g, 30 sec at 4 °C) keeping the bottom 75 μl and replacing the supernatant with equivalent volumes of fresh HbA (925 μl). Purified plasma membranes were pelleted using a final centrifugation at 16,000 × g for 20 minutes, resuspended in 10 ul HbA and immediately processed for Western blot.

### Immunoprecipitation

Immunoprecipitations were performed on oocytes injected with a mixture of cRNA coding for GFP-wt-AQP2 and untagged variants of AQP2, as specified in figure legends. Purified total membranes, as described above, were solubilized for 1 hour at room temperature using 2% deoxycholate in PBS then centrifuged at 100,000 × g for 30 min. Supernatants were first incubated with goat anti-GFP (1/1000 dilution, cat no.sc-5384, Santa Cruz Biotechnology) for 2 hours at room temperature under gentle agitation to which was added 20 μL pre-washed protein-G coated magnetic beads (dynabeads, Invitrogen) and further incubated for 30 mins. Beads were rinsed 4 times in solubilisation solution then dispersed in 40 μl Laemmli buffer, and heated at 95 °C for 5 min. Samples of 20 μl were loaded on gel and processed for Western blot detection. Also, 20 μl of starting material (initial solubilisations) were loaded in parallel to assess total AQP2 samples.

### Immunocytochemistry

Immunocytochemistry was performed on mpkCCD_c14_ cells grown on permeable filters and transfected with pBi-FLAG-AQP2 variants (wt and mutants) either in absence (single insert) or presence (double inserts) of untagged wt-AQP2 (24 hours incubation time). After treatment with forskolin (50 μM, 45 min^17^) in order to promote the plasma membrane targeting of AQP2, the cells were fixed at 4 °C for 20 minutes with paraformaldehyde (4%) diluted in PBS, and then rinsed in PBS. The immunolabeling was done using mouse anti-FLAG (1/1000 dilution, cat no: F3165, Sigma-Aldrich). Filters were mounted using an anti-quenching agent (Prolong Gold antifade, Molecular Probes, Eugene OR) prior visualization (60 X) using an Olympus IX-81 microscope along Image-Pro Plus v.5.0 software for images acquisition. Deconvolution was achieved using AutoQuant X2 software (MediaCybernetics).

### Western blots

Western blots were performed as described previously^31^ for both total (3 oocytes/sample) and purified plasma membranes (35 oocytes/sample). Samples were run on a 12% gel and transferred onto a nitrocellulose membrane. The efficiency of the overall procedure was monitored by Ponceau red staining. The membranes were first blocked with 5% non-fat milk in TBS-T (TBS + Tween 20, 0.1%) then incubated overnight at 4 °C with α-AQP2 (C-17, 1:1000, Santa Cruz Biotech, CA) followed the next day by incubation for one hour with secondary antibody (HRP-linked chicken anti-goat 1:25,000, Santa Cruz Biotech,CA). Blots were revealed using enhanced chemiluminescence detection (Phototope-HRP, New England Biolabs, Pickering, ON, Canada).

### Volume measurements

Functionality of AQP2 was evaluated by water flux measurements in water-injected and AQP2-injected oocytes. A detailed procedure is presented in a previous publication^32^. Briefly, the oocytes were placed in a 0.07 ml bath on the stage with the cross-section image monitored using an inverted, low powered microscope equipped with a camera and associated software to evaluate volume variations. A mild hypo-osmotic solution was applied to the oocytes by removing mannitol from the isotonic media (−20 mOsm). The variations in volume were used to determine water permeability values (P_f_) which are presented herein as % of *wt*-AQP2 activity.

### Data analysis

All experiments were performed at least three times using different oocyte batches or cell cultures and values are presented as mean ± SD. Oneway ANOVA was performed on all P_f_ determinations. Molecular weights and densitometry analyses were performed using software from alpha-imager 2000 (Alpha Innotech Corp, San Leandro, CA). Immunofluorescence data was gathered by measuring the number of positive cell (non-permeabiliezed fixation) in a random field along its adjacent 8 fields.

### Ethical approval

All procedures regarding protocols, manipulations and treatments of animals were approved and performed in accordance with both the Canadian guidelines and ethics committee of the Université de Montréal.

## Additional Information

**How to cite this article**: El Tarazi, A. *et al.* Functional Recovery of AQP2 Recessive Mutations Through Hetero-Oligomerization with Wild-Type Counterpart. *Sci. Rep.* **6**, 33298; doi: 10.1038/srep33298 (2016).
